# Annual Report of Surgeon-General of U. S. Army for 1873

**Published:** 1873-12-15

**Authors:** 


### MEDICAL EXAMINER.

*A Semi-Monthly Journal of Medical Sciences.*

EDITED BY

N. S. DAVIS, M. D., AND F. H. DAVIS, M. D.

Chicago, December 15th, 1873.

### EDITORIAL.

### ANNUAL REPORT OF THE SURGEON-GENERAL OF U. S. ARMY FOR 1873.

This is a pamphlet of only 13 pages, containing a statement of the financial operations of the medical department of our army during the year ending June 30th, 1873; the amount of sickness and mortality in the army during the same time; the number of specimens in the Army Medical Museum, and the additions thereto; the progress of the publication of the medical and surgical history of the late war; and some important recommendations in relation to filling vacancies in the Medical Corps, and an increase in the relative rank of medical officers. In regard to the last-named subject, the Surgeon-General says:

"I am compelled to again repeat the statement made in previous reports, that very serious and increasing injury has resulted to the service from the continued prohibition of appointments and promotions in the medical corps. The inducements of pay and rank, as at present established, are not sufficient to make the service attractive or remunerative to physicians already engaged in practice; and though, through the prerequisite rigid examination of candidates, it has heretofore been found possible to secure a high grade of talent and qualification, it is upon the younger portion of the profession, the recent graduates, that we must depend in filling up existing vacancies. As a large proportion of applicants fail to pass satisfactory examinations (which require in each case from three to six days), it will be the work of several years to restore the corps to the necessary standard of numbers as provided for in the act of Congress approved July 28, 1866. Although ambition to pass the Army Medical Board brings forward many of the most promising graduates of the medical colleges, additional inducements of rank, pay and promotion are becoming more and more necessary, not only to make the number of candidates equal to the needs of the service, but to retain the most desirable of them in the service under their frequent inducements to accept advantageous offers in civil life. The action of the American Medical Association, representing the medical profession of this country, regarding the unequal position of medical officers of the army—as compared to that of other staff corps—is based upon actual investigation of the subject, and presents to Congress all the facts in the case.

I can only urge most earnestly upon your attention the pressing and absolute necessity for such legislation as will secure to our officers and soldiers the efficient and reliable attendance in wounds and sickness which the Government should provide, and will make a position in the Medical Corps of the army, now, as formerly, an object of ambition to the best educated and best qualified young men in the profession."

The whole number of vacancies in the Army Medical Corps, is, at present, sixty-four; and we see no prospect of their being properly filled, unless Congress shall speedily add to existing inducements by providing increased rank and pay, after a fair term of faithful service. It would be well if every physician in the country who is acquainted with a member of Congress would urge upon him the necessity of passing the law that will be proposed for remedying this evil.

